# A longitudinal study on the information needs and preferences of patients after an acute coronary syndrome

**DOI:** 10.1186/s12875-016-0534-8

**Published:** 2016-09-20

**Authors:** Andrea Greco, Erika Rosa Cappelletti, Dario Monzani, Luca Pancani, Marco D’Addario, Maria Elena Magrin, Massimo Miglioretti, Marcello Sarini, Marta Scrignaro, Luca Vecchio, Francesco Fattirolli, Patrizia Steca

**Affiliations:** 1 Department of Psychology, University of Milan-Bicocca, Piazza dell’Ateneo Nuovo, 1, 20126 Milan, Italy; 2 Department of Medical and Surgical Critical Care, Cardiac Rehabilitation Unit, University of Florence and Azienda Ospedaliero, Universitaria Careggi, Florence, Italy

**Keywords:** Information needs, Acute coronary syndrome, Cardiovascular disease, Health information sources, Longitudinal research

## Abstract

**Background:**

Research has shown that the provision of pertinent health information to patients with cardiovascular disease is associated with better adherence to medical prescriptions, behavioral changes, and enhanced perception of control over the disease. Yet there is no clear knowledge on how to improve information pertinence. Identifying and meeting the information needs of patients and their preferences for sources of information is pivotal to developing patient-led services. This prospective, observational study was aimed at exploring the information needs and perceived relevance of different information sources for patients during the twenty-four months following an acute coronary syndrome.

**Methods:**

Two hundred and seventeen newly diagnosed patients with acute coronary syndrome were enrolled in the study. The patients were primarily men (83.41 %) with a mean age of 57.28 years (range 35–75; SD = 7.98). Patients’ needs for information and the perceived relevance of information sources were evaluated between 2 and 8 weeks after hospitalization (baseline) and during three follow-ups at 6, 12 and 24 months after baseline. Repeated measures ANOVA, Bonferroni post hoc tests and Cochran’s Q Test were performed to test differences in variables of interest over time.

**Results:**

Results showed a reduction in information needs, but this decrease was significant only for topics related to daily activities, behavioral habits, risk and complication. At baseline, the primary sources of information were specialists and general practitioners, followed by family members and information leaflets given by physicians. Relevance of other sources changed differently over time.

**Conclusion:**

The present longitudinal study is an original contribution to the investigation of changes in information needs and preferences for sources of information among patients who are diagnosed with acute coronary syndrome. One of the main results of this study is that information on self-disease management is perceived as a minor theme for patients even two years after the event. Knowledge on how patients’ information needs and perceived relevance of information sources change over time could enhance the quality of chronic disease management, leading health-care systems to move toward more patient-tailored care.

**Electronic supplementary material:**

The online version of this article (doi:10.1186/s12875-016-0534-8) contains supplementary material, which is available to authorized users.

## Background

Cardiovascular diseases (CVDs) are currently the largest single contributor to global mortality and will continue to dominate mortality trends in the future. Even though it is well known that modifiable factors related to lifestyle habits are major contributors to CVDs [1–5], patients generally fail to adhere correctly to medical advice or to change their unhealthy behaviors, causing continuous hospital readmissions [6, 7]. Literature has shown that the effective provision of appropriate health information is associated with better patient adherence to medical prescriptions [8], behavioral change [9, 10], increased patient satisfaction, reduced levels of psychological distress and enhanced perception of control over the disease [11–13]. Nevertheless, patients are often dissatisfied with the information that they receive, reporting unfulfilled needs in different areas related to disease care [14, 15]. In addition, patients’ needs are not always perceived correctly by health care providers [16, 17], compromising the quality and effectiveness of information provision. This situation may be due to insufficient knowledge on patients’ needs and, most importantly, on how those needs change over time. Evidence suggests that patients regarded all types of information as important, with a preference for three categories: medication, risk factors, and cardiac anatomy and physiology [18–24]. However, almost all of the studies used cross-sectional methods, which do not permit prediction of future need. One recent study has been conducted with the aim of investigating how patients’ information needs change over the course of a six-month long cardiac rehabilitation program, but it is still a cross-sectional study. It found that patients were significantly more interested in information about emergency/safety at the beginning of the program, while information about general/social concern and risk factors was preferred at the middle [25]. However, the adoption of a cross-sectional design is not appropriate for studying change over time. Other methodological weaknesses of these studies include small samples and the recruitment of participants at varied time points in the disease journey. Also, few studies included analysis of the relationships between needs and socio-demographic variables [26]. For instance, the role of gender is largely unexplored, although a few studies suggest that women want to be better informed and more active in the decision process than men [23, 27]. Women wanted more information overall, but also about specific topics such as angina and high blood pressure, whereas men wanted more information about sexual functioning [23].

Describing what information is needed for cardiac patients, from whom and at which point over the course of the disease is necessary to provide meaningful insight on how to enhance rehabilitation programs for two main reasons. First, the length of hospital stays for patients undergoing various cardiac procedures has decreased in recent years, especially for patients who have been treated for coronary heart disease [28, 29]. Actually, in the Italian health care system, patients with an episode of Acute Coronary Syndrome (ACS) are hospitalized for a period that can range from three to ten days (depending on the seriousness of the event). After discharge, patients follow an outpatient multifaceted and multidisciplinary rehabilitation program to improve functional capacity, recovery and psychological well-being [30, 31]. Rehabilitation program usually last for four weeks, during which patients attend physical activity sessions and educational courses with the aim to learn the role of behaviors in the management of CVDs. There are different follow-up medical examinations at one and six months after the ACS event, in which physicians assess the overall health status of the patient and check the pharmacological treatment making any appropriate changes (more other follow-up visits could be done in the following months depending on patient’s health situation). The shortened hospital stay and the few moments devoted to follow up examinations reduce health care practitioners’ opportunities to provide patients with health information. A second reason for why it is important to study the change in patients’ need and preferences refers to the psychological shock that is often experienced by patients after an acute cardiovascular event. The negative emotions (e.g., fear, anxiety, depression) related to the disease could limit patients’ abilities to absorb information during hospitalization and the days following the discharge. Due to the great importance of effective communication in chronic disease management and the lack of empirical evidence on what happens to need and preference for information over the course of disease, the aim of this study was to determine whether and how these variables change over time in a population of patients at their first ACS event. We also wanted to understand which information sources are perceived as most relevant in addressing health information. Research showed that physicians were preferred over nurses [26], while media sources like television or written material were less popular among cardiac patients [32]. However, there is no consensus in findings [19] and the methodological weaknesses reported above hold for this topic as well.

The research questions of the present study were as follows:

1. What are patients’ information needs and which information sources are perceived as more relevant over the twenty-four months following a cardiac event?
2. What are the information sources from which patients receive health information over the course of the disease?
3. What are the socio-demographic correlates of needs and perception of relevance of sources?

## Methods

### Participants and procedure

To be eligible for this study, subjects had to be newly diagnosed ACS outpatients involved in a rehabilitation program in one of three healthcare centers in Northern Italy; 30 years of age or older; able to speak and read Italian; without moderate-severe cognitive impairment, psychiatric disorders or diseases with limited expected survival.

Physicians at the hospital referred patients to the study and those who met the inclusion criteria were invited to participate. Between 2 and 8 weeks after hospitalization (baseline), patients were told about the aim of the study and were asked to sign an informed consent form. They were also informed about the three follow-ups at 6 (t1), 12 (t2), and 24 months after baseline (t3). Later a physician collected clinical data related to: a) ACS (Non-ST elevation myocardial infarction; ST elevation myocardial infarction, unstable angina); b) the revascularization procedure (i.e., percutaneous coronary intervention; c) anthropometric data; d) blood pressure values; e) the presence of CVD risk factors (hypertension, dyslipidemia, smoking history, diabetes, obesity, family history, physical inactivity) and f) pharmacological treatment. After the clinical examination, patients were asked to answer to a few questions by a trained researcher in order to measure their information needs and perceived relevance of information sources. This procedure was almost the same in all the follow-ups. The only difference was in the clinical examination: in fact, during the follows-ups further clinical information related to the number of a) specialist visits, b) emergency room visits c) new hospitalizations and d) new rehabilitation programs attended during the previous months were collected.

This study was approved by the Ethical Committee of the University of Milan-Bicocca and of the healthcare centers from which patients were recruited.

### Measures

#### Information needs

The information needs section comprised two questions evaluating the need for further information in one of six domains related to CVD management. These domains were:

- “Pharmacological Treatment”: information on the types of medicines to take, when to take them, and their possible interaction with other medicines;
- “Knowledge About the Disease”: information of the anatomical/functional nature connected to the disease (ex. how blood circulation system functions, what the symptoms connected to health problem are, and what can be done to manage them);
- “Daily activities”: information about daily life activities that can be carried out and which ones have to be modified (ex. work, free time, sexual activity);
- “Behavioral Habits”: information about the habits that can be continued and those that should be modified (smoking, diet, alcohol, physical activity);
- “Impact of the Disease”: information on how to manage stress and worries that might be generated by the change in life caused by the disease;
- “Risk and Complications”: information about the risks related to the disease and possible complications (ex. the possibilities of a heart attack, how to avoid complications, who to call in case of need etc.).

The first question was specifically designed to identify the amount of further information desired by the patients in these six domains (“*Indicate how much information you would like to receive about the following topics connected to the management of your cardiovascular disorders*”). The answer format was on a five-point Likert scale ranging from one (“I want to know nothing about the topic”) to five (“I want to know everything about it”). The second question asked patients to rate the importance of the six domains and to assign a value from 1-6 (*“Now please rate the importance of the topics listed below; you must assign a value from one for the most important topic, to six for the least important one)*. To identify the information needs, a balanced index was calculated by multiplying the score on question 1 by the score on question 2. Before computing the balanced index, the score on question 2 was reversed (for example, if a patient scored “1” on a topic, this response was recoded as six). The balanced index had a score range from 1-30 with higher scores indicating a higher need for that domain. This was calculated to control for patients’ tendencies to judge all information as “very” or “extremely” important. In fact, as found in previous research [26, 33], patients tend to report high scores when they are directly asked how much information they desire, and they may not be considering whether that information is useful for their specific situation. The balanced index provided a more accurate score of need for information rather than a general judgment of importance attributed to the topics.

### Information sources

Patients were first asked if they received information from a particular source (with a yes/no question). Then they had to evaluate, on a five-point Likert Scale ranging from one (“not at all”) to five (“very relevant”), the perceived relevance of the information received from the sources (“*Think about how you have learnt about your disease from the time you became aware you had the illness. For each of the sources listed below indicate how relevant it was in providing you with information*”). The sources were: “General Practitioner” (GP), “Specialist”, “Relatives”, “Friends”, “Information Leaflets given by Physician” (by the general practitioner or by the specialist), “Information Leaflets given by Associations” (such brochures could come from two main different providers: patients’ associations or organization, like the “Italian Association against Cardiovascular Disease”, or from commercial providers like pharmaceutical industry; in both cases, the brochure contain information about different aspects of the disease), “Magazines”, “Internet”, and “Television”.

### Socio-demographic variables

Participants were also asked to report general demographic information including their gender, age, marital and employment status, and education level.

The measures used in this study could be found as Additional file, in the Additional file section.

### Statistical analysis

Analysis of Variance (ANOVA) for repeated measures and Bonferroni post hoc tests were applied to test for differences in information needs and perceived relevance of sources over time. Cochran’s Q-test was used to compare the changes across the four time points (baseline, 6 months, 1 year, 2 years) for the dichotomous variable related to the proportion of patients that received health information from the different information sources. Regressions analyses were conducted to identify the relationship among socio-demographic variables (age, gender, marital status, employment and education level), information needs and the perceived relevance of sources. For all of the statistical analyses, a significant level was set at *p* ≤ 0.05. All analyses were performed using the Statistical Package for Social Sciences version 22.0 for Windows (SPSS Inc, Chicago, USA).

## Results

### Participants’ characteristics

The study included 217 patients with a mean age of 57.28 years (range 35–75; SD = 7.98), primarily male (83.41 %), married (70.96 %); 40.55 % with a high school degree, and mainly employed (58.06 %) (Table 1 reports full information about patients’ sociodemographic characteristics).

**Table 1:** Patients’ sociodemographic characteristics

Number of patients	217
Age, mean ± SD	57.28 ± 7.98
Gender	N (%)
Female	36 (16.58)
Male	181 (83.41)
Education	
< High School Diploma	106 (48.84)
High School Diploma	88 (40.55)
> High School Diploma	23 (10.59)
Employment	
Employed	126 (58.06)
Retired	58 (26.72)
Unemployed	11 (5.06)
Housewife	5 (2.30)
Retired with some work activities	17 (7.83)
Marital status	
Married	154 (70.96)
Not Married (Also widowed/divorced)	63 (29.03)

Regarding clinical data (Table 2), 71.88 % of the patients had ST elevation myocardial infarction (STEMI) and almost all had a percutaneous coronary intervention (94.01 %) with at least one stent (94.93 %). Roughly half (50.69 %) were affected by dyslipidemia, 29.95 % had family history of CVD, 16.59 % were affected by obesity and 17.97 % by diabetes.

**Table 2:** Patients’ clinical information

Acute coronary syndrome	N (%)
Non-ST elevation myocardial infarction (NSTEMI)	43 (19.81)
ST elevation myocardial infarction (STEMI)	156 (71.88)
Unstable Angina	18 (8.29)
Percutaneous coronary intervention	204 (94.01)
Patients with at least one stent	206 (94.93)
Risk factors	
Hypertension	97 (44.70)
Dyslipidemia	110 (50.69)
Smoking History^a^	140 (64.52)
Diabetes	39 (17.97)
Obesity	36 (16.59)
Family History	65 (29.95)
Physical Inactivity	17 (7.83)
	mean ± SD
Body Mass Index (BMI)	26.98 ± 4.11
Pulmonary Artery Systolic Pressure (PAS)	114.97 ± 13.37
Pulmonary Artery Diastolic Pressure (PAD)	72.71 ± 8.46

^a^This category includes patients who were smokers or have quitted less than twelve months before the baseline of the research

Further clinical information on the number of emergency room visits, new hospitalizations and new rehabilitation programs attended between the different follow-ups are reported in Table 3. In particular, in the months between baseline and t1, 8.29 % of patients visited the emergency room for chest pain, 4.14 % of patients were involved in ACS-related new hospitalizations, and 1.84 % of patients has attended a new rehabilitation programs. In the months between t1 and t2, 6.45 % of patients visited the emergency room for chest pain, 5.53 % of patients were involved in ACS-related new hospitalizations, and 4.14 % of patients have attended a new rehabilitation programs. Finally, in the months between t2 and t3, 5.99 % of patients visited the emergency room for chest pain, 4.15 % of patients were involved in ACS-related new hospitalizations, and 0.92 % of patients have attended a new rehabilitation programs.

**Table 3:** Emergency room visits, new hospitalizations and specialist visits in the follow-up

	6 months follow-up	12 months follow-up	24 months follow-up
	(T1)	(T2)	(T3)
	N (%)	N (%)	N (%)
Emergency room visits	18 (8.29)	14 (6.45)	13 (5.99)
New hospitalizations	9 (4.14)	12 (5.53)	9 (4.15)
Specialist visits	105 (48.39)	106 (48.84)	107 (49.31)
New rehabilitation programs	4 (1.84)	9 (4.14)	2 (0.92)

### Information needs

Regarding information needs, Mauchly’s test indicated that the assumption of sphericity had been violated for “Daily activities” (x^2^(5) = 12.99, *p* < .05) and “Behavioral Habits” (x^2^(5) = 17.36, *p* < .05). Therefore, the degrees of freedom were corrected using Greenhouse-Geisser estimates of sphericity (ε = .94 and ε = .95, respectively). The results from a repeated measures ANOVA showed a reduction in information needs over time for “Behavioral Habits”, “Risk and Complications” and “Daily activities”, while no reduction was found for the “Pharmacological Treatments”, “Knowledge About The Pathology” and “Impact of the Disease” domains. In all cases, the decrease was significant from baseline to t1, baseline to t2, and baseline to t3, but not between any other set of time points.

Table 4 presents the mean scores, standard deviation, test F and *p* levels.

**Table 4:** Information needs over time

Information need	Mean at baseline	Mean At	Mean At	Mean At	df	F
		6 months	12 months	24 months		
		follow-up	follow-up	follow-up		
		(T1)	(T2)	(T3)		
Pharmacological treatment	17.41	17.23	16.23	16.50	3;648	1.45
	(SD = 8.51)	(SD = 8.35)	(SD = 8.65)	(SD = 8.00)		
Knowledge About the Disease	18.01	17.69	17.16	16.75	3;648	1.29
	(SD = 8.77)	(SD = 7.96)	(SD = 8.18)	(SD = 8.13)		
Daily Activities	16.38	13.33	12.01	11.95	2.87;621.08	22.41**
	(SD = 8.28)	(SD = 7.14)	(SD = 6.86)	(SD = 7.37)		
Behavioral Habits	14.06	11.98	10.84	12.05	2.85;616.59	10.29**
	(SD = 7.87)	(SD = 7.17)	(SD = 6.70)	(SD = 7.13)		
Impact of the Disease’	13.26	12.08	11.92	11.59	3;648	2.67
	(SD = 7.57)	(SD = 6.71)	(SD = 7.16)	(SD = 6.77)		
Risk And Complications	18.70	16.99	16.62	15.91	3;648	6.41**
	(SD = 7.67)	(SD = .7.99)	(SD = 7.84)	(SD = 7.93)		

Note: ** Significant differences (*p* < .01)

### Information sources

A frequency analysis showed that patients reported receiving information immediately after their acute event, especially from “Specialists” (89.86 %), “GPs” (65.44 %), “Relatives” (79.26 %), “Friends” (67.74 %), and “Information Leaflets given by Physician” (62.67 %). Less than half of the sample received information from “Information Leaflets given by Associations”, “Magazines”, “Internet”, and “Television”. During the follow-ups, patients declared having received information from almost all sources in a greater extend compared to baseline (see Table 5). The Cochran’s Q test indicated that the differences in the provision of information from these sources over time were significant for: “Gp” (x^2^(3) = 59.45, *p* < .001); “Friends” (x^2^(3) = 9.62, *p* < .05); “Information Leaflets given by Physician” (x^2^(3) = 16.18, *p* < .001); “Magazines” (x^2^(3) = 14.49, *p* < .01); “Internet” (x^2^(3) = 9.64, *p* < .01); “Television” (x^2^(3) = 20.94, *p* < .001). For the other sources, no significant changes were found over time.

**Table 5:** Number and percentage of patients that have received information from a source over time

Information Sources	Baseline	6 months follow-up (T1)	12 months follow-up (T2)	24 months follow-up (T3)	df	Cochran’s Q
GPs	142 (65.44)	187 (86.17)	181 (83.41)	188 (86.63)	3	59.44***
Specialists	195 (89.86)	205 (94.47)	204 (94.00)	203 (93.54)	3	7.63
Relatives	172 (79.26)	186 (85.71)	170 (78.34)	178 (82.02)	3	4.33
Friends	147 (67.74)	161 (74.19)	139 (64.05)	132 (60.83)	3	9.64*
I. Leaflets-Physician	136 (62.67)	162 (74.65)	151 (69.58)	132 (60.83)	3	16.18***
I. Leaflets Associations	63 (29.03)	86 (39.63)	70 (32.25)	78 (35.94)	3	5.61
Magazines	90 (41.47)	116 (53.45)	112 (51.61)	113 (52.07)	3	14.49**
Internet	102 (47.00)	119 (54.83)	118 (54.37)	120 (55.29)	3	9.64**
Television	94 (43.31)	115 (52.99)	125 (57.60)	125 (57.60)	3	20.94***

Note: *Significant differences (*p* < .05); **Significant differences (*p* < .01); ***Significant differences (*p* < .001)

**Fig. 1:**
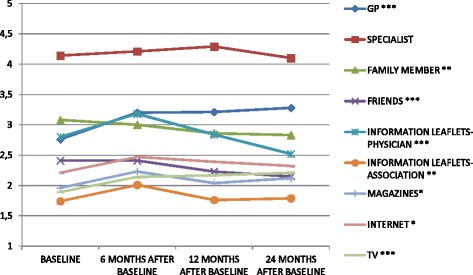
Relevance of different information sources over time. Note: *Significant differences (*p* < .05) ;** Significant differences (*p* < .01); *** Significant differences (*p* < .001)

Regarding relevance, Mauchly’s test indicated that the assumption of sphericity had been violated for all sources [“GP” (x^2^(5) = 25.11, *p* < .001), “Specialists” (x^2^(5) = 24.18, *p* < .001), “Family” (x^2^(5) = 24.63, *p* < .001), “Friends” (x^2^(5) = 16.42, *p* < .001), “Information Leaflets given by Physician” (x^2^(5) = 21.19, *p* < .001), “Information Leaflets given by Association” (x^2^(5) = 19.05, *p* < .001), “Magazines” (x^2^(5) = 14.69, *p* < .001), “Internet” ” (x^2^(5) = 21.01, *p* < .001), “Television” (x^2^(5) = 12.52, *p* < .001)]. Therefore, degrees of freedom were corrected using Greenhouse-Geisser estimates of sphericity (ε = 90, ε = 92, ε = 93, ε = 94, ε = 93, ε = 94, ε = 95, ε = 93, and ε = 96, respectively). A repeated measures ANOVA showed significant differences in the relevance of all the sources between the time points, except for “Specialist” (F(2.84, 613.20) = 2.26, *p* > .05); the direction of the change varied for the different sources (Fig. 1). In particular, the relevance of “GP” (F(2.78,600.02) = 15.39, *p* < .001), “Magazines” (F(2.88, 622.85) = 3.28, *p* < .05), “Internet” (F(2.87, 621.55) = 3.14, *p* < .05) and “Television” (F(2.89, 624.21) = 6.41, *p* < .001) increased over time, while it decreased for “Family” (F(2.77, 598.88) = 3.74, *p* < .01) and “Friends” (F(2.86, 618.42) = 6.04, *p* < .001). A particular trend appeared for “Information Leaflets given by physician” (F(2.84, 614.35) = 12.52, *p* < .001) and “Information Leaflets given by association (F(2.86, 618.76) = 3.82, *p* < .01): between baseline and t1 the relevance of these sources increased significantly, but it decreased significantly between the other sets of time points.

### The role of socio-demographics variables

At baseline, patients with higher education levels wanted less information about “Pharmacological Treatments” (*β* = -.191, *p* < .01) and more information about “Daily activities” (*β* = .142, *p* < .05) and “Behavioral Habits” (*β* = .194, *p* < .01). Age was related to “Distress” (*β* = -.181, *p* < .05), with older patients less interested in information on how to manage stress related to the disease. Gender was related to the need on information on “Risk and complications” (*β* = .136, *p* < .05), with female patients more interested in this topic.

When the relationships between information needs and demographic variables were analyzed over time, the need for information on “Daily life activities” resulted associated with patients’ gender (F(2.87; 592.28) = 3.30; *p* = .020) and marital status (F(2.87; 592.28) = 2.70; *p* = .045), with female and married patients who desired more information on this topic over time. The need for information on “Distress” was positively associated with marital status (F(3; 618) = 3.64; *p* = .013), employment (F(12; 318) = 1.92; *p* = .029) and age (F(3; 618) = 4.65; *p* = .003).

Regarding information sources, age and gender were significantly related to the perceived relevance of “GPs”; in particular, older (*β* = .228, *p* < .01) and male patients (*β* = -.149, *p* < .05) perceived that the information provided by this source was more relevant Age was also positive related to the perceived relevance of “Family” (*β* = .252, *p* < .01) and “Television” (*β* = .351, *p* < .001). Gender was positively related to the perception of relevance of “Information Leaflets given by Associations” (*β* = .166, *p* < .05), with female patients who perceived information from this source as more relevant. Patients with higher education levels perceived that the information from the “Internet” was more relevant (*β* = .177, *p* < .01), while patients with lower education levels perceived sources such as “Family” (*β* = -.233, *p* < .001), “Information Leaflets given by Physician” (*β* = -.212, *p* < .01), “Information Leaflets given by Associations” (*β* = -.183, *p* < .01), and “Television” (*β* = -.185, *p* < .01) were more relevant. Employment was related only to the perceived relevance of “Television” (*β* = -.222, *p* < .01), with patients retired or unemployed who relied more on this source.

Results from repeated measure Anova showed that gender, age and marital status were related to the perception of relevance “Gp”, “Family”, Friend”, and “Information Leaflets given by Associations”. Gender was positively associated with relevance of “Gp” (F(2.76; 570.09) = 2.72; *p* = .043). The perceived relevance of “Gp” resulted also associated also with age (F(2.76; 570.09) = 2.82; *p* = .038), with older patients who relied more on this source. Marital status was positively related with the perceived relevance of “Family” (F(2.79;575.81) = 3.54; *p* = .014), “Friends” (F(2.86; 589.61) = 4.89; *p* = .002), and “Information Leaflets given by Associations” (F(2.86; 589.29) = 2.67; *p* = .047); married patients declared to perceive these sources as more relevant as the disease progresses.

## Discussion

This longitudinal study aimed to gain understanding of how the information needs and perceptions on sources of health information of patients with ACS may change over time in order to be able to better tailor information giving. To our knowledge, this study is among the first to quantitatively investigate in the ACS setting information needs and perceptions over time and which characteristics are related to a possible change in needs and perceptions.

A reduction in information needs in all six domains related to disease management was observed, but the decrease was significant only for daily activities, behavioral habits, and risk and complication. For information related to drugs, knowledge about the pathology and on how to manage the distress, a non-significant decrease was revealed.

A possible explanation of the overall decrease of patients’ needs is that immediately after their first cardiac event patients desire all of the information they can get to cope with the new situation and to manage the distress caused by the illness and the cardiac procedures they have undergone. Then, over time, this desire decreases with a gradual reduction in the disease symptoms and greater experience with the condition [21, 34, 35]. Another explanation could be related to the transformation of the patients’ role during the course of the disease. At the beginning patients play a more passive role in the management of their situation; they are located in a controlled place and must comply with medical direction. After discharge and over the following months patients become more active, choosing on what information to focus their attention. It is important to note that the two domains directly related to self-management of the disease, daily activities and behavior, are the topics patients wanted to be less informed about over time, while they continued to desire “medical” information about drugs and pathology. This preference for medical information over information about lifestyle has been shown in previous research [16, 36, 37] and deserves attention by health practitioners. It could be supposed that patients don’t want information on daily life activities or behavior because they already have correct habits. However this supposition is not sustained by research, which shows that patients fail to adhere correctly to medical advice or to change their unhealthy behaviors, causing continuous hospital readmissions [38].

Socio-demographic characteristics were partially associated with information needs and with their changes over time. In particular, being married was significantly associated with needing more information on behavioral habits and distress over time. This could arise from the fact that, in addition to their own worries and anxiety, patients have to manage their families’ distress. Spouses usually do not participate in the educational programs offered by hospitals and they may feel incapable of providing optimal support to their sick partners. As a consequence they could increase patients’ anxiety by their own apprehension. Perhaps patients’ high information needs also reflect partners’ needs for information on topics on which they could have a primary role. The informational topic related to the management of stress and worries caused by the disease was the one which was most related to sociodemographic variables over time; in fact, it was associated not only with marital status, but also with age and employment, with older patients who do not work (both for retirement and unemployment) who desire less information at the beginning of the disease and over time. These relationships seem to underline that younger patients are more aware of the dangerous role of distress for health and they experience a deeper burden related to the disease in comparison with older patients. Patients’ education level was significantly associated with information needs only at the beginning of the disease. In particular, highly educated patients wanted less information about pharmacological treatments and more information about daily activities and behavioral habits. This could reveal greater knowledge about the treatment as well as a greater understanding of their crucial role in the disease management. Other relationships arose at baseline between being female and desire more information on daily life activities and risk and complications of the disease. This result is in line with some evidences in research, which suggest a non-participatory role adopted by men and older patients in the management of their illness and a greater desire for information in women [23, 39, 40]. However, there is a lack of consensus in research on the role of socio-demographic variables in explaining patients’ needs [33].

Regarding information sources, both the repeated measures Anova and the Cochran Test show similar results. Patients reported having received information from GPs in a higher amount over time and perceiving this source as more relevant during the follow-ups. These results may be explained by an increase in the number of consultations over the course of the disease. In fact, it is unlikely that immediately after a cardiovascular problem patients interact with a GP, except for drug prescriptions. After the discharge patients tend to consult the specialist less and mainly refer to the GP for any advice on disease management. The relevance of the specialist does not increase over time, most likely because of a ceiling effect: patients indicated high scores for the specialist at baseline, making it impossible to score significantly higher on the follow-ups. The relevance of “Magazines”, “Internet”, and “Television” increased over time; in addition, the results show that patients received significantly more information from these sources as the time passed, maybe due to a more active role of the patients after discharge; it is possible that in the first few weeks/days after the heart attack, they follow the lead of specialists without actively searching for autonomous additional information. Then, with a reduction in the medical examinations, patients may pay more attention to other sources. However, it is important to note that the scores for these sources, together with information leaflets given by associations and friends do not reach the 2.5 level, indicating that these sources are not perceived to be highly significant. Regarding family and information leaflets given by physicians, their relevance decreases after twelve months, showing that these sources are perceived as mildly relevant only at the beginning and in the few months following the disease. Surprisingly, we have not observed the Internet to be of great importance in informing patients about health, even though there is a significant increase in the use of this source. Maybe the perceived lower relevance of the Internet could be explained by the characteristics of our sample: patients participating in this study had a mean age of 57 years, and more than 26% of them were retired; they may thus not be familiar with computer and Internet use and prefer to use simpler sources to gather information. This hypothesis is confirmed by the significant relationship between education level and the perceived relevance of this source. In addition, Italy lags behind other countries in the use of the Internet [41] and this could be a further explanation of this result.

Even for the relations among sociodemographic variables and sources of information, multiple patterns of relationships emerged at the beginning of the disease and over time. In particular, at baseline, age, gender and education influenced patients’ perceptions, with male, elderly and low educated patients who rely more on the information provided by general practitioners, family members, television, and information leaflets. Some relationships between sociodemographic variables and patients’ perceptions remain significant over time; in particular, marital status was related with the perceptions of relevance of friends, family and information brochures, with married patients that perceive these sources as more relevant as the disease progresses. Such patients seem to prefer “traditional” ways to obtain information, like direct discussions with physicians or other people, written take-home information, or videotapes, maybe because these sources are more reassuring and allows them to take some control over their health [23].

The definition of how patient needs and perceptions change during the course of a chronic disease provides information upon which to inform and guide future research and facilitate information provision based on the preferences set by the patient, relevant to their individual situation and context. These findings present an opportunity for healthcare practitioners (HCPs) to be aware of their patients’ information needs and be able to recognize how much information their patients may require in each moment of the disease.

As such different recommendations emerge:

- currently, HCPs invest much effort in information giving at the initial moment of an ACS. From a secondary prevention point of view, the results from this longitudinal study suggest that it is also necessary to shortly explore the patients’ information needs at every follow up visit and not only during the hospitalization for an acute cardiovascular event. In particular, it is important to constantly provide patients with information about pharmacological treatment, clinical aspects connected to the disease and recommendations on how to better manage stress and worries generated by the disease;
- furthermore, HCPs should deepen the underlying reasons of the constant decrease of the need for information related to behavioral habits. If this decrease is a consequence of patients’ incomprehension of the importance of lifestyle in maintaining health, it is important to find new and more fitting ways to deliver this kind of information. In particular, it could be rethought the role of general practitioners; the results of this study, in fact, show that the relevance of this source of health information increase over time. GPs should be provided of instruments (primarily the appropriate time) to better guide patients in the self-management of the disease;
- although magazines, internet and television were scored as less relevant in comparison with other sources, their increased relevance over time suggests a greater participation and interest of patients in the process of obtaining information. This result could be used by HCPs as an incentive to use these sources as powerful tools in delivering health information.

### Limitations

The limitations of this study mainly pertain to three aspects. First, the sample was composed of patients involved in cardiovascular rehabilitation (CR). CR is a comprehensive outpatient risk reduction program that aims to improve capacity and quality of life giving patients the tools to optimally self-manage their condition. During CR, patients attend multiple courses that involve lifestyle habits as well as psychosocial factors. It is possible that exposure to CR affected patients’ perception of the importance in disease management of specific health domains influencing their needs. Therefore, the results here presented may not apply to patients who do not participate in CR. Second, 85% of participants were men. Although heart disease is often considered a problem for men, CVD is the second-leading cause of death in women 45 to 64 years of age [42]. Thus, further research must more deeply investigate information needs using a sample in which the number of men and women are equalized. Third, in the study here presented, only patients’ socio-demographic variables were considered as possible factors correlated to needs and preferences. Future studies might consider using other features like patients’ health literacy and psychological characteristics such as locus of control or coping as key factors associated with needs and preferences.

## Conclusion

The information needs and the perceptions on different sources of information of patients with ACS change as the disease progresses. Taking into account these changes is pivotal to develop patient-led services and to enhance the quality of chronic disease management, leading health-care systems to move toward more patient-tailored care. Findings from this study have the potential to inform and guide HCPs, in particular general practitioner which are perceived ad more relevant as the time passes, on how to better provide health information to patients throughout the entire course of a chronic disease.

## Additional files

Additional file 1: Study measures. (PDF 178 kb) Additional file 2: DATABASE. (XLS 191 kb)

## Abbreviations

ACS: Acute coronary syndrome
ANOVA: Analysis of Variance
CR: Cardiovascular rehabilitation
CVD: Cardiovascular disease
GP: General practitioner
HCPs: Healthcare practitioners
SPSS: Statistical Package for Social Sciences
