# Supplementary material for: A longitudinal study on the information needs and preferences of patients after an acute coronary syndrome
Source: BMC Fam Pract. 2016 Sep 20;17:136. doi: 10.1186/s12875-016-0534-8 (PMC5028970; doi:10.1186/s12875-016-0534-8)
Supplement: Additional file 1: — Study measures. (PDF 178 kb) [file 12875_2016_534_MOESM1_ESM.pdf]

MEASURES

THE INFORMATION NEEDS SECTION

Baseline (t0) instructions:

Please answer the following questions, concerning the information that you are given and that you would like to get in relation to your acute coronary syndrome.

Six (t1), twelve (t2), twenty-four (t3) months follow-up instructions:

Please answer the following questions, concerning the information that you are given from your last visit to today and the information that you would like to get in relation to your acute coronary syndrome.

Please indicate how much information you would like to receive about the following topics connected to managing your acute coronary syndrome:

	Not at all<br>1	2	3	4	Very much<br>5
**A – PHARMACOLOGICAL TREATMENT**:<br>information on the types of medicines to take, when to take them, and their possible interaction with other medicines, etc.	☐	☐	☐	☐	☐
**B – KNOWLEDGE ABOUT THE DISEASE**:<br>information of an anatomical /functional nature connected to my disease (ex. how my blood circulation system functions, what the symptoms connected to my health problem are, and what I can do to manage them etc..)	☐	☐	☐	☐	☐
**C – DAILY LIFE ACTIVITIES:**<br>information about my daily life activities that I can continue to carry out and about which ones I should modify (ex. work, free time, sexual activity, etc.)	☐	☐	☐	☐	☐
**D – BEHAVIOURAL HABITS**:<br>information about the habits that I can continue and those that I should modify (ex. smoking, diet, alcohol, physical activity etc.)	☐	☐	☐	☐	☐
**E – THE IMPACT OF THE DISEASE**:<br>information as to how to manage stress and worries that might be generated by the change in my life caused by this disease.	☐	☐	☐	☐	☐
**F – RISKS AND COMPLICATIONS**:<br>information about the risks related to my disease and possible complications (ex. the possibilities of a heart attack, how to avoid complications, who to call in case of need etc.)	☐	☐	☐	☐	☐

Now please rate the topics listed below, giving 1 (to the first topic in order of importance) to 6 (to the last topic in order of importance)

TOPIC	ORDER OF IMPORTANCE
A – PHARMACOLOGICAL TREATMENT	◯
B – KNOWLEDGE OF THE DISEASE	◯
C – DAILY LIFE ACTIVITIES	◯
D – BEHAVIOURAL HABITS	◯
E – IMPACT OF THE DISEASE	◯
F – RISKS /COMPLICATIONS	◯

THE INFORMATION SOURCES SECTION

Baseline (t0) instructions:
Think about how you have learnt about your acute coronary syndrome from the time you became aware of having the illness. For each of the sources listed below indicate if it provided you with information about acute coronary syndrome.

Six (t1), twelve (t2), twenty-four (t3) months follow-up instructions:
For each of the sources listed below indicate if it provided you with information about acute coronary syndrome in the previous months (from your last visit to today).

	**YES** 1	**NO** 0
a. General Practitioner/family doctor	☐	☐
b. Specialist	☐	☐
c. Family members	☐	☐
d. Friends /acquaintances	☐	☐
e. Information leaflets etc. given out by the family doctor or the specialist	☐	☐
f. Information leaflets etc. given out by associations	☐	☐
g. Magazines and books	☐	☐
h. Internet	☐	☐
i. Television programs	☐	☐

For each of the sources listed below indicate how relevant it was in providing you with information about acute coronary syndrome.

	**Not at all** 1	2	3	4	**very** 5
a. General Practitioner/family doctor	☐	☐	☐	☐	☐
b. Specialist	☐	☐	☐	☐	☐
c. Family members	☐	☐	☐	☐	☐
d. Friends /acquaintances	☐	☐	☐	☐	☐
e. Information leaflets etc. given out by the family doctor or the specialist	☐	☐	☐	☐	☐
f. Information leaflets etc. given out by associations	☐	☐	☐	☐	☐
g. Magazines and books	☐	☐	☐	☐	☐
h. Internet	☐	☐	☐	☐	☐
i. Television programs	☐	☐	☐	☐	☐
